# Proliferative Fasciitis of the Hand in a Nine-Year-Old Girl: A Case Report and Review of the Literature

**DOI:** 10.7759/cureus.6763

**Published:** 2020-01-24

**Authors:** Marco Preziosi, Nicolas De Saint Aubain, Maria P Aparisi Gómez, Paolo Simoni

**Affiliations:** 1 Radiology, Queen Fabiola Children's University Hospital, Brussels, BEL; 2 Pathology, Institut Jules Bordet, Brussels, BEL; 3 Radiology, Auckland City Hospital, Auckland, NZL

**Keywords:** sarcoma soft tissue

## Abstract

Proliferative fasciitis (PF) of the hand is a rare condition, which typically occurs in adulthood. To date, only two dozen cases of PF have been reported in children. This benign condition can mimic malignant soft tissue tumors such as soft tissue sarcoma. We present a case of histopathologically confirmed PF of the fifth right finger in a nine-year-old girl, along with the imaging workup. We also provide a review of the previous literature on PF in children.

## Introduction

Proliferative fasciitis (PF) is a benign pseudosarcomatous myofibroblastic proliferation of the soft tissues [1]. In most cases, PF is a rapidly-growing and self-limiting process [2]. However, in children, PF does not usually undergo spontaneous regression. PF usually presents as a painful soft tissue mass, located in the subcutaneous adipose tissue, without trespassing the muscular aponeurotic superficial fascia. Clinically, PF can mimic soft tissue sarcoma [2]. The histological appearance of PF has been extensively described in the literature; mainly in case reports due to its rarity. We present radiographs, ultrasound (US), and MRI findings correlated with histopathological specimens in this case report on a nine-year-old girl with PF of the right hand.

## Case presentation

**Figure 1:**
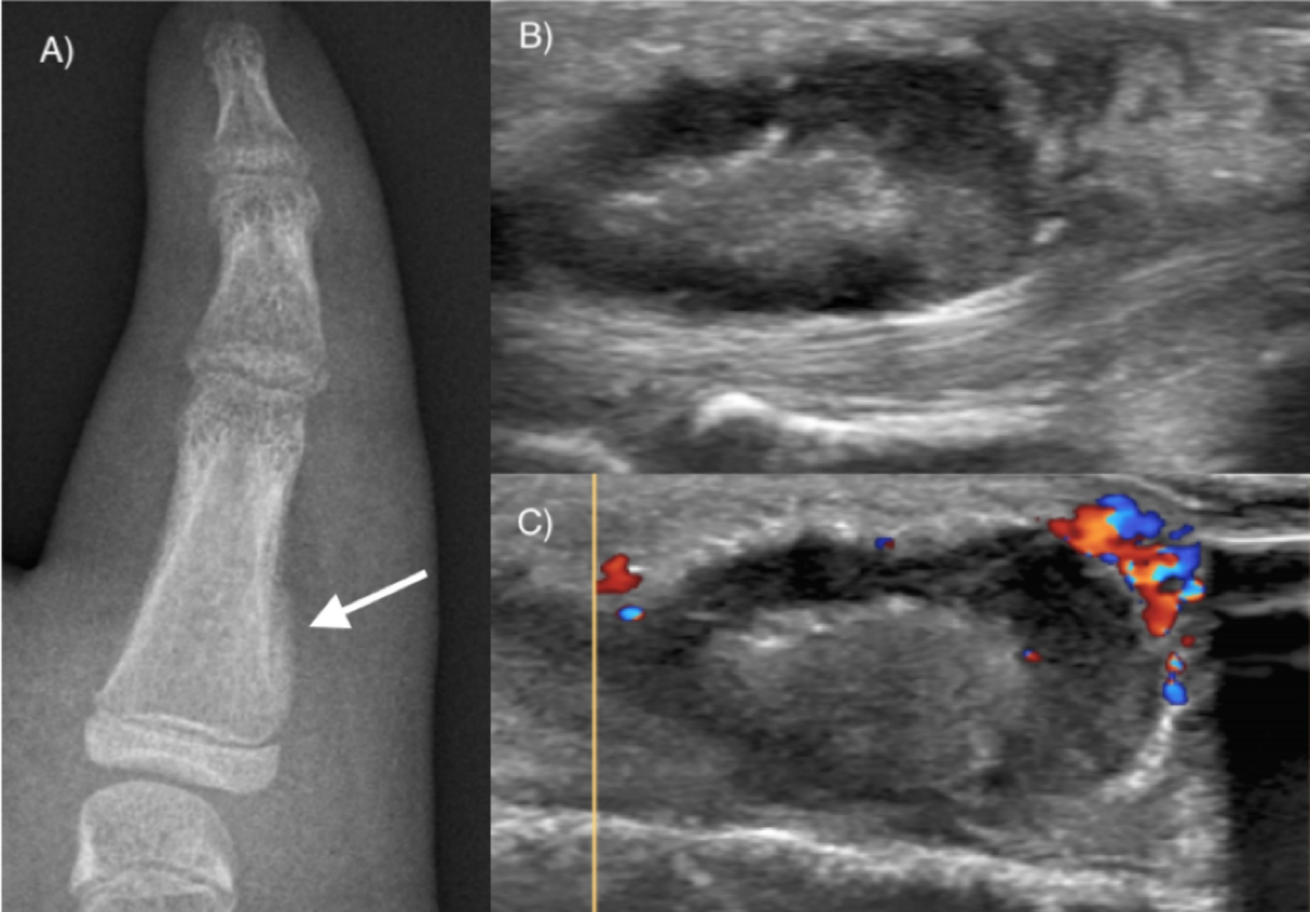
Representative radiologic and ultrasonographic findings A) radiograph demonstrating periosteal reaction (white arrow) in the ulnar aspect of the proximal phalanx of the fifth finger; B) ultrasound B-mode imaging shows a central echogenic region, with a hypoechoic rim; C) Doppler interrogation demonstrates minimal vascularity associated with the hypoechoic region, as well as surrounding hyperemia

An otherwise healthy nine-year-old girl presented to the emergency department with pain and swelling around the fifth right finger metacarpophalangeal (MCP) joint, and a palpable mass. Symptoms had appeared a week before consultation and progressed since then with a rapid increase in size. There was no history of recent trauma or fever in clinical records. At clinical examination, the subcutaneous mass was firm, not adhered to the skin, and tender to palpation. There were no inflammatory signs. The mass was located in the ulnar aspect of the fifth MCP joint. Laboratory findings were unremarkable, with normal inflammatory markers. A plain radiograph of the right hand showed a periosteal reaction in the ulnar aspect of the proximal phalanx of the fifth finger. Ultrasound revealed a 22-mm oval mass located in the palmar aspect of the fifth MCP joint, contiguous to the fifth flexor tendons and surrounded by diffuse soft tissue edema. The lesion presented an echogenic central area, with no visible signal on color Doppler interrogation, and a hypoechoic rim with mild vascularization (Figure 1).

**Figure 2:**
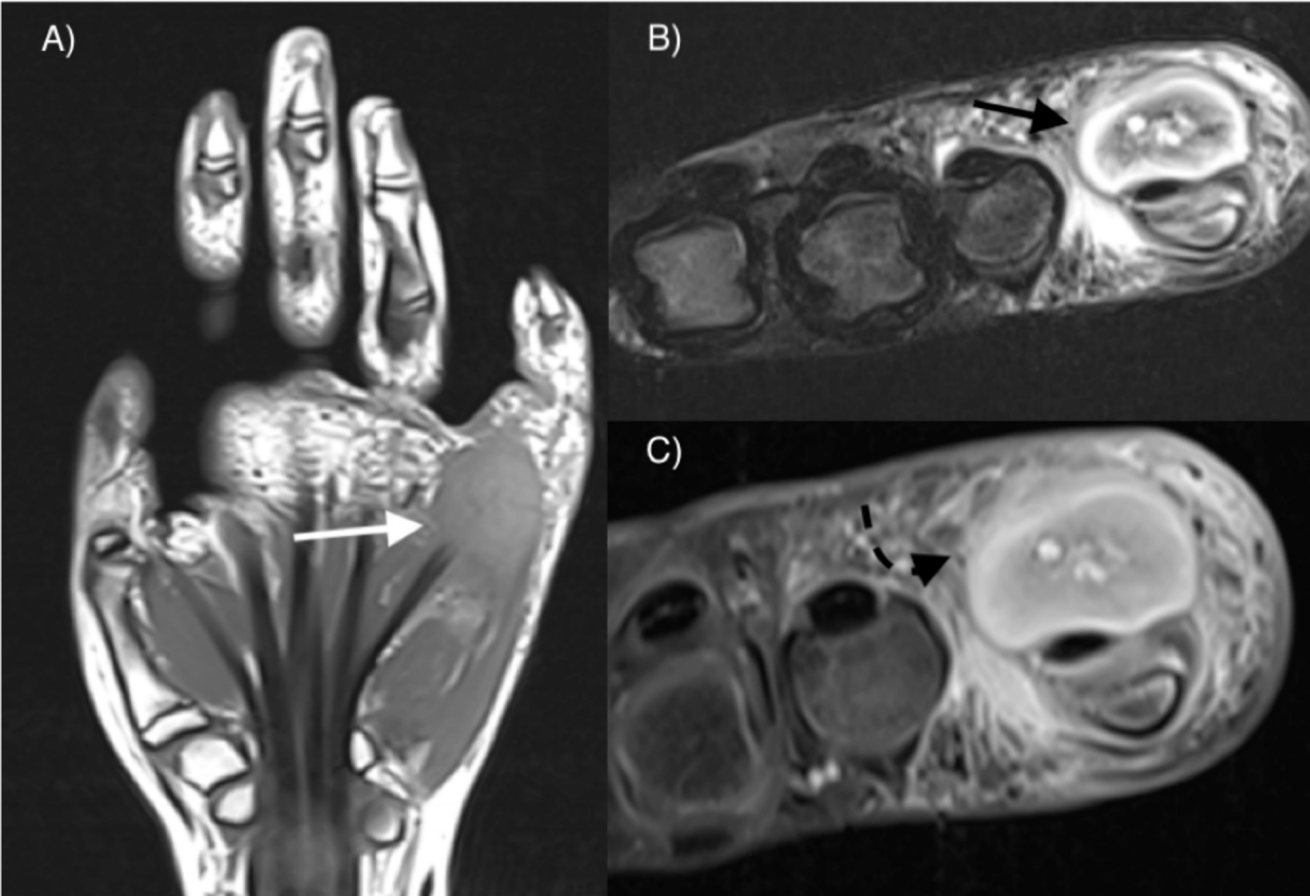
MRI findings A) coronal T1-weighted demonstrates a mass which is homogeneously isointense to muscle (white arrow); B) axial T2-weighted FS demonstrates heterogeneous signal, hyper-intense in the periphery (black arrow); C) contrast-enhanced axial T1-weighted FS image demonstrates rim-like enhancement (curved discontinuous arrow), as well as enhancement of some central foci MRI: magnetic resonance imaging; FS: fat suppression

**Figure 3:**
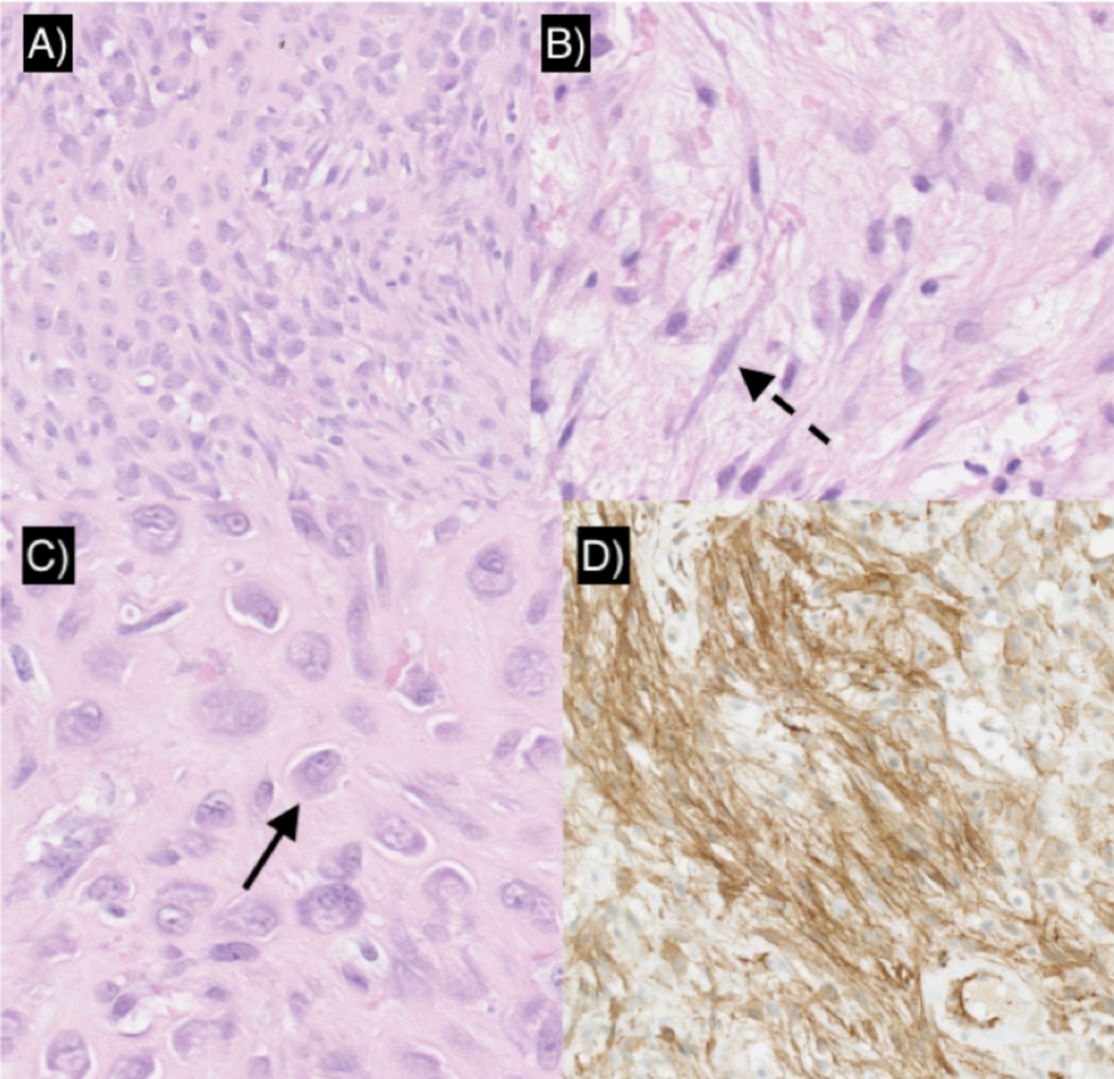
Histopathological and immunohistochemical findings A) sheet-like arrangement of ganglion-like myofibroblasts areas mingled with loose spindle-like cells in a myxoid and collagenous matrix; B) view on spindle-like cells (discontinuous black arrow); C) ganglion-like cells (magnification x 50) (black arrow); D) immunostained slide (magnification x 50) showing diffuse positive cellular stain for smooth-muscle actin (SMA), more intense for spindle cells. The stain for desmin and myogenin are negative instead

The MRI revealed a soft tissue mass, located on the outer surface of the muscular superficial fascia. The mass was homogeneously isointense compared to muscle on T1-weighted images and demonstrated heterogeneous signal on T2-weighted fat-suppressed (FS) images, more intense in the periphery than in its central zone. FS T1-weighted images after contrast administration demonstrated thin rim enhancement, and also enhancement of small regions within the mass, corresponding to microvascular zones with high T2 signal (Figure 2). No hemorrhage or calcification was visible within the lesion on T2 images. Peripheral soft tissue edema and moderate tenosynovitis of the flexor tendons were also noted. Based on imaging work-up, rhabdomyosarcoma (RMS) was suspected, and a core biopsy was performed. Histopathological and immunohistochemical results were consistent with PF, a benign pseudosarcomatous myofibroblastic proliferation (Figure 3). A ‘wait and see’ approach was adopted, based on the several cases of self-limiting PF in adults described in the literature. However, an MRI follow-up three months later showed no regression, and the lesion was excised.

## Discussion

PF was first described by Chung and Enzinger in 1975 [1]. It is a type of mass-forming subcutaneous proliferation currently included in the heterogeneous group of benign myofibroblastic proliferation, which also includes nodular fasciitis (NF) and proliferative myositis (PM). PF is a lesion typical of the adult population, mainly seen in patients between 40 and 70 years of age, with no ethnic-group predilection. PF is considered a self-limiting condition in adults. Unlike in adults, no evidence of spontaneous involution was observed in a series of 20 cases reported in children [2]. PF is quite uncommon in patients younger than 15 years of age. Most cases of PF occur in the subcutaneous tissue of the extremities, and most frequently in the forearm [3]. 

The genetic background of PF is still unknown. A clonal aberration (MYH-USP6 gene fusion) was reported only for NF, suggesting a benign neoplastic origin [4]. This mutation has not been described in PF, which is therefore still considered as a reactive myofibroblastic proliferation. It has been suggested that minor trauma or chronic inflammation may trigger PF. However, any history of preceding injury has rarely been reported in the literature in connection with PF, raising the possibility of other causes [1,3].

PF can mimic sarcomas such as RMS and epithelioid sarcoma (ES) because of its rapid growth and histological features. A giant cell tumor of the tendon sheath (GCTTS) could also be included in our differential diagnosis, because of the particular location of the lesion, which is contiguous to the fifth flexor tendon. Early lesions show components of increased cellularity and myxoid change that can mimic the usual findings in NF [5]. However, the absence of hemosiderin deposition on MRI (a hallmark of GCTTS) [5] and the biphasic cell population were not consistent with this diagnosis in our case. The differential diagnosis of pediatric PF may also include other rare entities such as reticulohistiocytoma, xanthogranuloma, and epithelioid hemangioendothelioma (EH) [5]. 

Because the imaging features of PF are non-specific, diagnosis often relies on pathological findings. The diagnosis of PF must be confirmed histopathologically. Like in NF, it shows a tissue-culture proliferation of spindle myofibroblasts in a variably myxoid and collagenous stroma [3,6]. The histologic hallmark of both PF and PM (differing only in location) is the additional presence of large, polygonal ganglion-like myofibroblasts characterized by large vesicular nuclei, prominent nucleoli, and abundant basophilic-amphophilic cytoplasm (Figure 3C). Those ganglion-like myofibroblasts, along with the spindle myofibroblasts make up the characteristic biphasic cell population of PF (Figures 3B; Figure 3A). Some histologic differences have been described between the most common adult form and the rare pediatric form. Pediatric PF is usually more lobular than infiltrating (contrary to the adult form). In children, PF presents higher cellularity, higher mitosis rate, higher myxoid/collagen ratio, and a more solid growth pattern than in adults. Also, pediatric PF can present regions of acute inflammation and/or necrosis; these features are usually not found in the typical adult form [7]. The pathogenesis of PF is controversial, and the genetic background is unknown.

The immunohistochemical findings are similar to those of NF and PM. The spindle-shaped cells stain for SMA (Figure 3D). Immunostains for cytokeratins, S-100 protein, desmine, myogenin, and B-catenin are usually negative. The ganglion-like cells may also stain for actin even if less consistent [3,8]. Differential diagnosis of pediatric PF should include sarcomas, such as RMS and ES [7]. RMS has rhabdomyoblasts similar to ganglion-like cells, but the tumor cells in our case did not demonstrate cross-striations at histology or ultrastructural skeletal features [9]. Spindle cells in RMS display nuclear atypia, consistently absent in PF [3,6]. Moreover, desmin and myogenin, usually positive in RMS, were absent in our immunostains [6]. 

The classic type of ES can fit in the differential diagnosis of pediatric PF. Like PF, ES has tumor cells with abundant cytoplasm, areas of necrosis or inflammation and spindled cells. Unlike ES, PF is typically negative for cytokeratins and does not show *SMARCB1* deletion or mutations [6]. 

## Conclusions

We reported a case of a nine-year-old girl with PF. PF is a rare disease in children. When present in children, it exhibits some similarities and some differences with the adult form. Pediatric PF should be considered in the differential diagnosis when dealing with a superficial soft-tissue mass in children, to avoid misdiagnosis of the malignancy. Imaging can help to narrow the differential diagnosis and to plan the biopsy and surgery, although the differentiation between PF and a malignant soft-tissue mass, such as RMS, requires a histopathological examination.
